# Possible gasoline-induced chronic liver injury due to occupational malpractice in a motor mechanic: a case report

**DOI:** 10.1186/s13256-017-1352-x

**Published:** 2017-07-03

**Authors:** Mahesh Lakmal Gunathilaka, Madunil Anuk Niriella, Nathasha Vihangi Luke, Chathura Lakmal Piyarathna, Rohan Chaminda Siriwardena, Arjuna Priyadarshin De Silva, Hithanadura Janaka de Silva

**Affiliations:** 1 University Medical Unit, Colombo North Teaching Hospital, Ragama, Sri Lanka; 2 Department of Medicine, Faculty of Medicine, University of Kelaniya, PO Box 6, Thalagolla Road, Ragama, GQ 11010 Sri Lanka

**Keywords:** Hydrocarbon, Gasoline, Occupational liver injury, Chronic liver injury, Cirrhosis, Case report

## Abstract

**Background:**

Hydrocarbon-induced occupational liver injury is a well-known clinical entity among petroleum industry workers. There are many types of hydrocarbon exposure, with inhalation being the most common. Hydrocarbon-induced occupational liver injury is a rarely suspected and commonly missed etiological agent for liver injury. We report a case of a non-petroleum industry worker with chronic liver disease secondary to hydrocarbon-induced occupational liver injury caused by chronic low-grade hydrocarbon ingestion due to occupational malpractice.

**Case presentation:**

A 23-year-old Sri Lankan man who was a motor mechanic presented to our hospital with decompensated cirrhosis. He had been chronically exposed to gasoline via inadvertent ingestion due to occupational malpractice. He used to remove gasoline from carburetors by sucking and failed to practice mouth washing thereafter. On evaluation, he had histologically proven established cirrhosis. A comprehensive history and workup ruled out other nonoccupational etiologies for cirrhosis. The patient’s long-term occupational gasoline exposure and clinical course led us to a diagnosis of hydrocarbon-induced occupational liver injury leading to decompensated cirrhosis.

**Conclusions:**

Hydrocarbon-induced occupational liver injury should be considered as a cause when evaluating a patient with liver injury with possible exposure in relevant occupations.

## Background

Hydrocarbons are organic substances composed of carbon and hydrogen molecules. They are classified as aliphatic, in which carbon moieties are arranged in chains, or aromatic, in which the carbon atoms are arranged in a ring. Halogenated hydrocarbons make up a subgroup of aromatic hydrocarbons in which one hydrogen atom is replaced by a halogen molecule [1]. Gasoline, kerosene, lubricating oil, mineral spirits, and organic solvents are commonly encountered hydrocarbons in day-to-day practice. Gasoline is a refined product of petroleum consisting of a complex mixture of aliphatic and aromatic hydrocarbons, additives, and blending agents. Additives and blending agents are added to the hydrocarbon mixture to improve the performance and stability of gasoline.

There are many work activities associated with hydrocarbon exposure. Hydrocarbons in various forms are used in industrial processes such as paint manufacturing, automobile manufacturing and maintenance, and metal processing. Exposure to these toxins can be intentional or accidental, and the routes of exposure may be inhalational, owing to their being highly volatile, or via direct contact and absorption through the skin. A wide range of health hazards related to short-term and long-term exposure to hydrocarbons have been identified. Pulmonary complications, especially aspiration, are the most frequently reported adverse effect of hydrocarbon exposure [2].

Toxic hepatitis is a well-recognized complication, especially following exposure to halogenated hydrocarbons [3]. The main pathogenic mechanisms responsible for liver damage caused by organic substances are inflammation, dysfunction of cytochrome P450, mitochondrial dysfunction, and oxidative stress [4]. Because the liver is the site of detoxification for most of these substances, it is exposed to the harmful effects of these toxins. Occupational toxic hepatitis can be divided into three types: hepatocellular, cholestatic, and mixed. Liver disease is likely to be more severe in the hepatocellular type, and elevated bilirubin in hepatocellular injury indicates serious liver disease [4]. Patients with cholestatic or mixed type are likely to develop chronic disease more frequently than those with hepatocellular type [4]. The common histopathologic pattern in toxic liver injury is centrilobular necrosis (zone III) necrosis. Liver biochemistry can be altered within 24 hours after ingestion of a toxic substance.

Although a number of organic substances are known to cause liver injury resulting from occupational exposure, hydrocarbon-related occupational liver injury (HC-LI) remains an underdiagnosed entity [5]. Three conditions must be fulfilled to diagnose toxic liver injury resulting from occupational exposure: (1) Liver damage should occur after occupational exposure to the toxic substance; (2) liver enzymes must increase to at least double the upper limit of normal; and (3) other causes of liver disease should be excluded [6].

Very little is known about the frequency and patterns of HC-LI. It is difficult to assess liver damage due to hydrocarbons in the workplace, owing to difficulties in quantifying exposure [7]. Therefore, individual case reports or case series on HC-LI are still valuable sources of knowledge. We report a case in a motor mechanic with HC-LI following chronic ingestion of gasoline resulting from occupational malpractice.

## Case presentation

A 23-year-old Sri Lankan man, who had been working as a motor mechanic for approximately 5 years, presented to our hospital with a 2-month history of progressive, yellowish discoloration of the eyes; bilateral ankle swelling; and abdominal distention not associated with right hypochondrial pain, pruritus, or fever. His past medical history was unremarkable except for being prescribed furosemide by a general practitioner for edema. He denied intake of any other drugs, health supplements, or herbs. He had no history of smoking and only occasionally consumed alcohol in safe amounts (within Asian standard <14 U/week). His last alcohol consumption had been several months prior to the onset of symptoms. He had no family history of chronic liver disease. He had worked in a garage as a motor mechanic and used to remove gasoline from carburetors by sucking using his mouth when suction pumps were not available. He did not practice proper mouth washing and used to swallow petroleum compounds in substantial amounts during most of his working days. He had continued this malpractice for nearly 5 years until he developed symptoms.

**Fig. 1:**
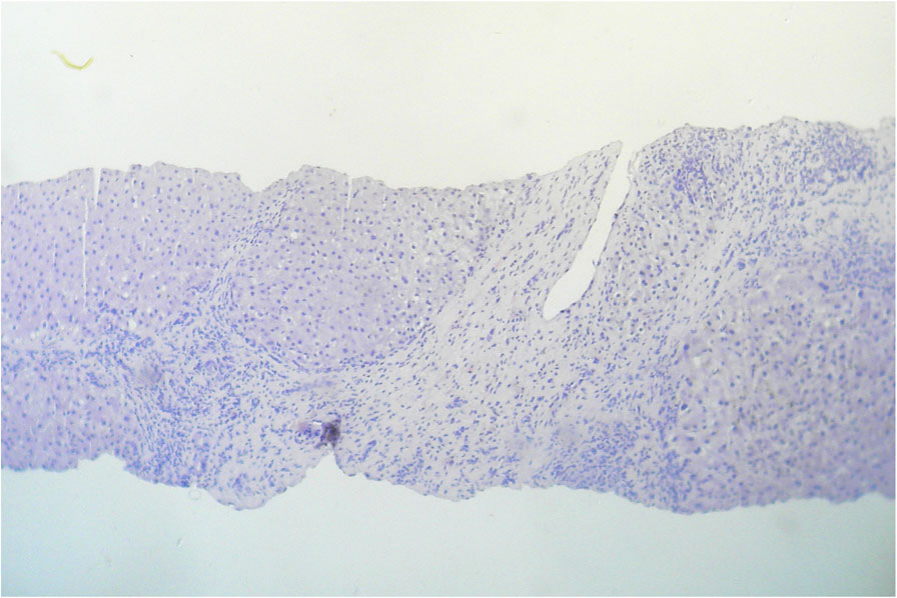
Liver biopsy showing features of cirrhosis (hematoxylin and eosin stain, original magnification ×40)

His clinical examination revealed icterus and ankle edema with nontender hepatomegaly and moderate ascites. The results of the rest of his clinical examination were normal. His laboratory data were as follows: hemoglobin 12.2 g/dl, white blood cell count 8920/mm^3^, platelet count 204,000/mm^3^, erythrocyte sedimentation rate 16 mm at the end of the first hour, aspartate aminotransferase 190 U/L, alanine aminotransferase 45 U/L, alkaline phosphatase 465 U/L, γ-glutamyl transpeptidase 72 U/L, total bilirubin 26.4 mg/dl (direct bilirubin 12.8 mg/dl), total protein 58.4 g/L (albumin 23.2 g/L, globulin 35.2 g/L), prothrombin time 19.8 seconds (international normalized ratio 1.67), and serum creatinine 0.9 mg/dl. His laboratory results were negative for anti-hepatitis A virus (HAV) immunoglobulin M antibody, anti-HAV immunoglobulin G antibody, hepatitis B surface antigen, anti-hepatitis C virus antibody, human immunodeficiency virus (HIV) antibody and antigen, antinuclear antibody, anti-smooth muscle antibody, and antimitochondrial antibodies. His levels of serum ferritin, serum copper, and serum ceruloplasmin were within normal ranges. The result of slit-lamp biomicroscopy was normal. Abdominal ultrasonography revealed hepatomegaly with a coarse hepatic echotexture suggestive of cirrhosis, portal hypertension, and moderate ascites without focal liver lesions. Esophagogastroduodenoscopy revealed small esophageal varices with portal hypertensive gastropathy. His liver biopsy revealed evidence of cirrhosis with regenerating hepatocytes, but with no clue to the underlying etiology of his liver disease (Fig. 1).

The patient was managed conservatively with symptomatic treatment. His regular medications included ursodeoxycholic acid, spironolactone, furosemide, lactulose, and carvedilol. His symptoms and laboratory parameters gradually improved during his hospital stay and stabilized over several weeks (Table 1). He was able continue his regular occupation with cautious handling of petroleum compounds. Given his advanced Child-Turcotte-Pugh (CTP) class C (CTP score 10/15) and high Model for End-Stage Liver Disease score (25), he is currently being worked up for a live-donor liver transplant.

**Table 1:** Liver biochemistry of patient

	On admission	On day 3	On discharge	6 weeks postdischarge
ALT, U/L	52	39	35	36
AST, U/L	205	160	80	55
Total bilirubin, mg %	26.41	21.17	10.35	1.97
ALP, U/L	465	365	253	123

*Abbreviations: ALT* Alanine aminotransferase, *AST* Aspartate aminotransferase, *ALP* Alkaline phosphatase

## Discussion

Our patient, a 23-year-old, previously healthy motor mechanic, presented to our hospital with decompensated cirrhosis. His history revealed chronic occupational exposure to gasoline via the oral route due to a malpractice in cleaning vehicle carburetors. A comprehensive workup ruled out other etiologies for cirrhosis. His liver biopsy confirmed established cirrhosis. His long-term occupational gasoline exposure and clinical course led us to a diagnosis of possible HC-LI leading to decompensated cirrhosis. Although not conclusively proven in this case, in the absence of other etiology and owing to our patient’s chronic exposure to a well-known hepatotoxic agent with a compatible time course, we were led to the possibility of a clinical diagnosis of gasoline-induced chronic liver injury.

Numerous studies have suggested exposure to hydrocarbons, especially halogenated hydrocarbons, as a cause of hepatotoxicity [8, 9]. Our patient fulfilled all diagnostic criteria for HC-LI [6]. The unique route of exposure through chronic low-grade ingestion and the patient’s occupation indirectly related to exposure to petroleum products differentiated the present case from previously reported cases of HC-LI. HC-LI is rarely diagnosed, because it is not commonly suspected [5]. Therefore, taking a detailed history and maintaining a high index of clinical suspicion are needed for the diagnosis of HC-LI. This case highlights the consequences of occupational malpractice-related HC-LI, which can easily be prevented by health education among potentially exposed workers in high-risk work environments.

## Conclusions

Chronic exposure to gasoline leading to HC-LI should be considered as an etiological factor for the development of cirrhosis in workers in occupations with possible exposure. Taking a detailed occupational history is necessary to establish HC-LI because this entity is underestimated and commonly missed in clinical practice. Because many young people in the economically productive age group are engaged in high-risk occupational activities, we suggest increasing awareness regarding mishandling of petroleum-based products to prevent the development of HC-LI and to reduce the burden on health care systems.

## Abbreviations

ALT: Alanine aminotransferase
AST: Aspartate aminotransferase
ALP: Alkaline phosphatase
CTP: Child-Turcotte-Pugh
HAV: Hepatitis A virus
HC-LI: Hydrocarbon-related occupational liver injury
